# A Comprehensive Study on Cyber Attacks in Communication Networks in Water Purification and Distribution Plants: Challenges, Vulnerabilities, and Future Prospects

**DOI:** 10.3390/s23187999

**Published:** 2023-09-20

**Authors:** Muhammad Muzamil Aslam, Ali Tufail, Ki-Hyung Kim, Rosyzie Anna Awg Haji Mohd Apong, Muhammad Taqi Raza

**Affiliations:** 1 School of Digital Science, Universiti Brunei Darussalam, Gadong BE1410, Brunei; 21h8565@ubd.edu.bn (M.M.A.); ali.tufail@ubd.edu.bn (A.T.); rosyzie.apong@ubd.edu.bn (R.A.A.H.M.A.); 2 Department of Cyber Security, Ajou University, Suwon 16499, Republic of Korea; 3 Department of Electrical and Computer Engineering, The University of Massachusetts Amherst, Amherst, MA 01003, USA; taqi@umass.edu

**Keywords:** IIoT, Industry 4.0, water industry, cyber security, attacks, challenges and future prospective

## Abstract

In recent years, the Internet of Things (IoT) has had a big impact on both industry and academia. Its profound impact is particularly felt in the industrial sector, where the Industrial Internet of Things (IIoT), also known as Industry 4.0, is revolutionizing manufacturing and production through the fusion of cutting-edge technologies and network-embedded sensing devices. The IIoT revolutionizes several industries, including crucial ones such as oil and gas, water purification and distribution, energy, and chemicals, by integrating information technology (IT) with industrial control and automation systems. Water, a vital resource for life, is a symbol of the advancement of technology, yet knowledge of potential cyberattacks and their catastrophic effects on water treatment facilities is still insufficient. Even seemingly insignificant errors can have serious consequences, such as aberrant pH values or fluctuations in the concentration of hydrochloric acid (HCI) in water, which can result in fatalities or serious diseases. The water purification and distribution industry has been the target of numerous hostile cyber security attacks, some of which have been identified, revealed, and documented in this paper. Our goal is to understand the range of security threats that are present in this industry. Through the lens of IIoT, the survey provides a technical investigation that covers attack models, actual cases of cyber intrusions in the water sector, a range of security difficulties encountered, and preventative security solutions. We also explore upcoming perspectives, illuminating the predicted advancements and orientations in this dynamic subject. For industrial practitioners and aspiring scholars alike, our work is a useful, enlightening, and current resource. We want to promote a thorough grasp of the cybersecurity landscape in the water industry by combining key insights and igniting group efforts toward a safe and dependable digital future.

## 1. Introduction

IoT has limited processing capability and a low-power network of sensing devices that interchange data with each other, such as cloud servers and gateways, usually via wireless or wired technologies. The use of IoT devices is increasing, and it is estimated that by the end of 2030, the number of connected devices will reach around 24.1 billion [1]. However, progress in the IoT has highlighted security concerns. Considering the variety of IoT topologies and the abundance of sensing and communication modules they incorporate, integrating such devices leads to a dynamic and varied landscape [1,2]. Actually, IoT devices are considered and, in some cases, known to be vulnerable to network attacks, such as spoofing, data theft, denial of service attacks (DDoS attacks), and phishing attacks [3]. According to a lab study, nearly 30% of attacks on IoT devices occur on wireless or mobile technology devices [4]. In several situations, IoT device security is handled by the device’s own application [5]. For instance, it has been shown that battery-powered wearable, handheld, and portable gadgets frequently sacrifice security performance in order to support longer working times. Although energy-efficient communication and control protocols for IoT have been developed to address these problems, vulnerabilities associated with these protocols may still be present. Bluetooth Low Energy (BLE) affects a wide range of IoT devices and is widely used for industrial IoT (IIoT) and wearable devices. Basically, IIoT is the extension of IoT in industry. Focusing on machine learning, machine-to-machine (M2M) communication, big data, and IIoT-capable enterprises, industries will have suitable reliability and efficiency in operation. The interaction of operational technology (OT) and information technology (IT) marks a distinction between IoT and IIOT [6,7].

IIoT systems are more vulnerable to advanced persistent threat (APT) adversary assaults than conventional industrial control systems (ICS) and OT networks because of IT network connectivity and the introduction of M2M, which connects different intelligent and latest devices. In addition, ICS devices often work on legacy (without a security mind) or unaware (no updates from time to time) software. Attackers or “Evil Eyes” usually target ICS-related information to gather intelligence that can shut down critical systems, endanger human lives, and disrupt industrial processes. In the past, such attacks have occurred in the real world; hence, we can say they were real-world attacks (RWA). Examples include Iran’s uranium Stuxnet attack in 2010 that caused a sufficient setback to Iran’s nuclear program and damaged their centrifuge [8]; the German steel mill attack in 2014 that caused significant loss (the mill avoided blast burning from shutting down) [9]; the power company attack in Ukraine in 2015 that detached small-level stations from the power grid, leaving millions of customers without power for a long time [10]; the similar attack in Ukraine in 2016 on transmission-level substations that disconnected capital without power for more than an hour [11]; and the attacks on the petrochemical plant in Saudi Arabia in 2017 that shut down the safety controller of the plant which could have caused massive loss, damage, or an explosion [12]. Industry, the malware behind the power grid attack in Ukraine in 2016 and the Saudi petrochemical attack in 2017 are still active in targeting energy sectors and electrical power substations [13,14]. In-controller IIoT malware that was recently revealed in 2022 consists of modules that hit particular ICS devices such as Schneider Electric PLCs using Modbus, open platform communication (OPC) servers, Codesys protocols, and Ormon PLCs.

**Figure 1:**
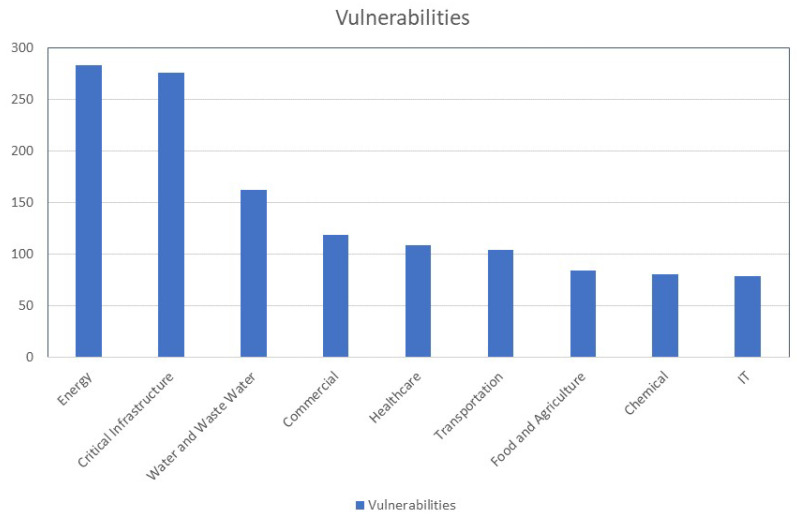
Cyber attacks in various industries [29] (vulnerabilities).

The energy, critical manufacturing, water/wastewater, and commercial sector are the major sectors affected by cyber attacks, as shown in flow chart Figure 1, while the US Department of Home Security considers the water and waste industry to be the main target of cyber attacks [15]. Securing it [16,17] is considered to be a national priority [18]. From 2012 to 2015, this industry received the most assessments from the Cybersecurity and Infrastructure Security Agency Industrial Control Systems Cyber Emergency Response Team [18]. Recently, in August 2022, a criminal attack on a UK water company was observed which gained access to customer banking details [19]. Cybersecurity has developed a huge interest in the water industry and some educational programs have been offered [20,21]. This provoked great interest in the research environment [22,23,24,25,26,27], as earlier cybersecurity incidents may have provided insightful information that could be used to inform current cyber defense efforts, promoting investments and initiatives and increasing their applicability and efficiency. This calls for a thorough compilation and analysis of these instances, which is currently lacking.

**Figure 2:**
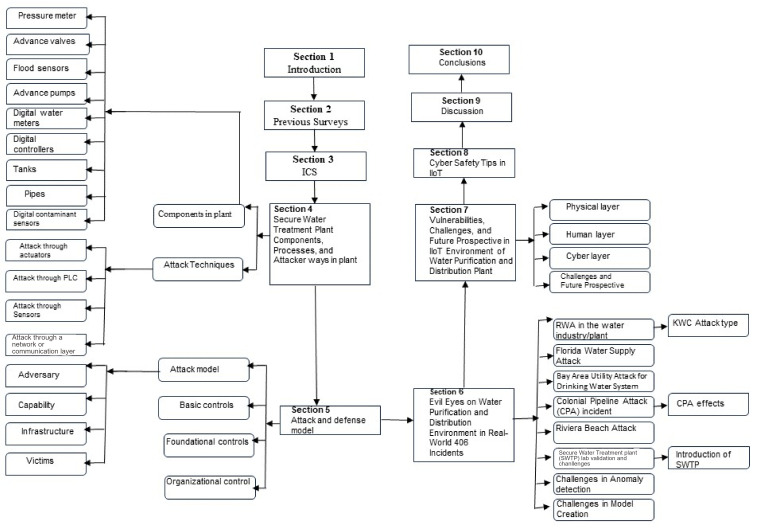
Paper architecture.

This study presents a study of water industry vulnerabilities and security, a review of malicious, documented, and disclosed cybersecurity attacks in the water industry, and safety guidelines or tips. This review also presents the challenges and future prospective analyses from real-world water industry attacks. Figure 2 represents the paper’s architecture. This study highlights the attacker’s role and “proof” in water purification and distribution plants. According to a study in 2021, the average instance of cybersecurity and data breaches will eventually be 15% yearly [28]. As of the time at which we wrote this review, there has been no research that provides a comprehensive study of security issues, vulnerabilities, and real-world cases in water distribution and purification plants. This review is new, and we hope to maximize researchers interest.

The rest of the paper has been organized as follows: Section 2 concerns the comparative evaluation of previous surveys. In Section 3, we discuss the architecture of the industrial control system. Section 4 contains a discussion of secure water treatment plant components and processes, as well as the methods by which an attacker could gain access to a plant. In Section 5, we discuss the attack and defense model in the ICS environment. Section 6 concerns attacks on water purification and distribution plants in real-world scenarios. In Section 7, we discuss the vulnerabilities, challenges, and future of water purification and distribution plants, while Section 8 concerns cyber saftey tips in IIot. In Section 9, we discuss security in water purification and distribution plants, and our conclusions are given in Section 10.

## 2. Previous Surveys

IoT is a technology that links physical devices through a wired or wireless network, and IIoT is a technology that deals with complicated architecture and is linked with the hardware and software in the industry. Actuators and sensors play an important role in data transmission and communication in an ICS environment such as industrial architecture. The authors of [30] studied IoT problems and challenges, but their work is limited, and the authors did not explain the applications of IoT or the role of IoT in the industrial domain. IoT-based applications have been studied in [31], in which the authors presented a general review of IoT-based applications. This work is useful for researchers in understanding the IoT applications and the advancement of the technology, but the authors did not discuss security issues or future perspectives in this work. Moreover, very few IoT applications were discussed. IoT advancement from various perspectives such as social impacts, applications, and challenges has been studied in [32,33]. The work is far from a discussion of security issues and future challenges in IoT. A review has been presented in [34] which is IoT-based but limited to healthcare applications. Future research on green IoT has been discussed in [35]. With the development of technology and IoT’s role in connectivity, industrial IoT (IIoT) has been playing an important role in academia and industry. The authors of [36,37] presented a review on IIoT, and they explained the interaction of IoT with IIoT. A security review of IoT has been discussed in [38], but the authors did not present their work from the perspective of applications and cases. The authors of [39] studied emerging technologies in the IIoT, and their work explained the experimental and conceptual architecture of IIoT. IIoT has been reviewed from a business perspective in [40].

Security is a key issue in an ICS environment, and researchers are doing their best to identify and overcome these issues. The authors of [41] presented a review of security in ICS. Research shows that there is insufficient attention being paid to the patch management process because of SCADA complications. The authors presented several security issues and SCADA assessments, but in this work, the authors were to able to highlight maximum security issues in an ICS environment and solutions. An ICS environment covers several applications, such as the chemical industry, oil and gas, water and wastewater treatment, agriculture, and many others. Our focus is on cyber security in water and wastewater treatment plants.

Water is a basic element of life. It is considered one of the major sectors affected by cyberattacks. Researchers’ contributions are playing an important role in raising awareness of such issues. The role of IoT in water distribution systems has been studied in [42]. The authors of [43] presented a review of cyber security in water treatment plants, and they also discussed real cases in water treatment plants. The authors discussed fifteen real-life cases in the water purification and distribution industry. The authors discussed the attacker’s methods and vulnerabilities in the system, but their work did not explain the latest cyberattack incidents in the water industry, and there is also insufficient discussion of the future prospects in the water and wastewater industry. The authors of [22] presented a systematic review of cyber security in the critical infrastructure of water and wastewater treatment plants. The authors discussed future research work, but they did not study real cases and the attackers’ means of entering the water plant’s communication network. Their work also did not explain the key challenges and vulnerabilities in the system. A study has been presented in [44] that explains the water distribution [45] system while ignoring the security aspects involved in distribution. Cyberattack mitigation in an ICS environment has been studied in [46]. The authors of [47] presented a review of cyber attacks and detection methods in water purification plants. The survey is based on general cyber attacks and common attack detection methods and it does not cover the latest research and incidents in water purification plants. The work also fails to present the vulnerabilities in the system.

These surveys show insufficient knowledge of cyber security in water distribution and purification plants. There are recent incidents in water purification and distribution plants that have not been discussed in these surveys. Moreover, attacker entranceways and system vulnerabilities have not been discussed in previous surveys. Very few of the previous works dedicated space to future prospects, and none covered the subject extensively. At the time of writing this manuscript, there has been no survey on attack detection in water purification and distribution plants. In previous surveys, significant information has been missing regarding the security of water purification and distribution plants. The current survey will therefore be very useful and informative, offering maximum information and knowledge for researchers and industrialists. We provide a comparison with previous surveys in Table 1.

## 3. Industrial Control System (ICS)

In this section, we provide work on the integration of IT with traditional OT networks and the general architecture considered for ICS networks. We will also discuss adhering to this ICS architecture when we discuss real-world incidents in water treatment plants.

Usually, an ICS architecture consists of software and hardware components, which are known as supervisory control and data acquisition (SCADA) for systemic monitoring, process control, ICS device communication, data collection, log data storage, and system monitoring. In a typical SCADA system, the lowest level consists of dumb devices, also called field devices, such as sensors, actuators, etc. Remote terminal units (RTU) and programmable logic controllers (PLC) are used to control these devices. Generally, RTU and PLC are computer-based systems that give control signals to field devices, obtain data, and send it to central control stations such as the master terminal unit (MTU). The MTU communicates in a master-slave model via a telephone network, wireless or wired network, or the internet for monitoring the field devices, uploading fresh or updated configurations, and sending commands. All such operations are managed by the human–machine interface (HMI) that is linked to the MTU and permits gathering data, transferring signals to remote sites, and varying configurations and settings.

**Figure 3:**
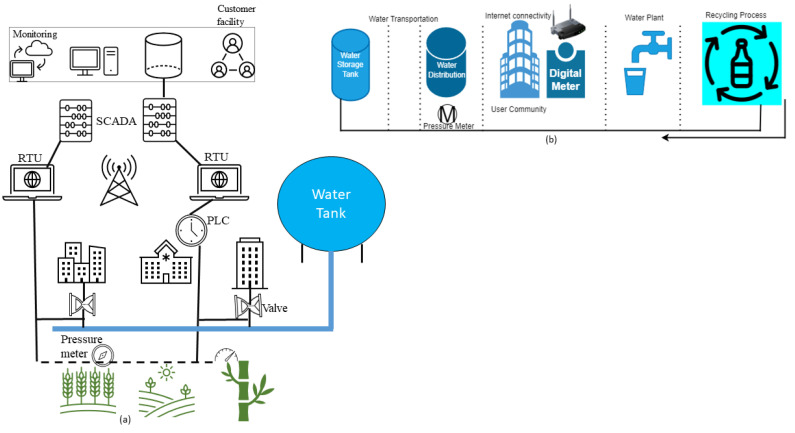
(**a**) Old architecture of a water distribution system. (**b**) Architecture of a water purification and distribution system.

An old water distribution system architecture is shown in Figure 3a with PLC and RTU distributed sites. In this simple network, we discussed various layers of SCADA architecture in which field devices such as pressure gauges and valves are under the monitoring of RTU through a wireless system, while the role of SCADA is that of a central control station, such as the head of the ICS network, and it is in communication with PLC and RTU in the environment.

Previously, there was an air gap between the SCADA system and traditional OT system networks, which were not interlinked with the IT system. With the advancement of technology, several industries moved to overlap the IT and OT network, which is known as the cost-effective approach. On the one hand, this approach is considered cost-effective and time-saving; on the other hand, there seems to be serious security threats involved in this approach, for the following reasons:

- There are different operational priorities of OT and IT networks, such as availability vs. confidentiality, and both may not adjust;
- Several ICSs lack security features such as access control and data encryption;
- There are limited visualization options for implementing and assessing security adjustments, which are provided by expensive legacy devices in ICS setups;
- The immediate adoption of corrective measures that may require system interruptions is prohibited by the critical and real-time business activities in OT as well as safety rules.

**Figure 4:**
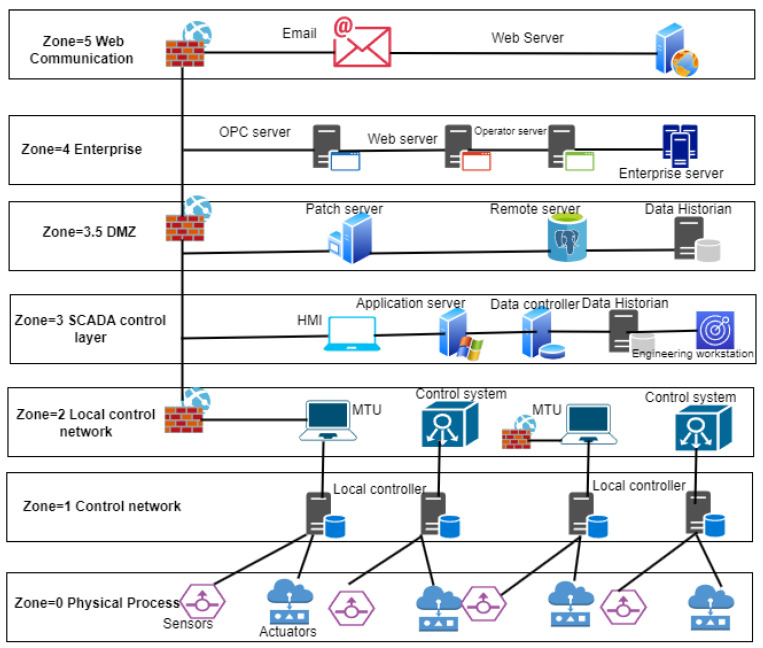
An ICS architecture.

As a result of these security measures, some limitations have been suggested by security experts for access to the OT network. There are some other works in the ICS security field intended to enhance protocols, standards, and components to support security benefits. In an ICS, the development of technology has merged the IT and OT networks, which is known as IIoT and is without air gaps. Figure 4 is an ICS network architecture that consists of various zones and can be described as an industrial automation and control system security. Basically, a zone is defined as a set of devices or components (IT and OT) joined together for a subclass of applications in an ICS environment.

## 4. Secure Water Treatment Plant Components and Processes, and Attacker Means of Access

IIoT-deployed water plants or systems increase with the development of technologies such as monitoring and sensing technology and real-time communication technology such as satellite and wireless communication, intelligence, and controls.

### 4.1. Components in Plant

There are various components used in water purification plants. These are given as follows.

#### 4.1.1. Pressure Meter

This is used to detect a pressure leakage in the plant such as via flooding, pipe burst, or any other kind of irregularity. It senses and stops the flow or transportation of water.

#### 4.1.2. Advance Valves

With the variation of environmental situations, these stop or regulate the water flow in the system. Smart valves permit bidirectional water flow and stop the reduction of water or signal the water meter to reduce the oversupply of water.

#### 4.1.3. Flood Sensors

These analyze the existing and impending safety with respect historically occurring and impending flood disasters. They stop the flow of water when they detect a pipe break in a source to stop water loss.

#### 4.1.4. Advance Pumps

To pump water via pipelines into and out of tanks, these machines need electricity. They permit water to flow both ways and are employed for high-energy-use processes and can stop a significant waste of water.

#### 4.1.5. Digital Water Meters

These are electrical devices that assess water consumption on a regular basis. Data on water use by consumers are gathered by the water utility administration from the smart water meters via communication networks. The information gathered is then utilized to calculate the cost of water consumption and manage bills.

#### 4.1.6. Digital Controllers

These have thermostats for sprinkler systems that allow water to be automatically irrigated based on schedules or specific circumstances, which is useful in preventing wastage of water and energy occurring due to excess use of water.

#### 4.1.7. Tanks

These are used for the purpose of storing water received from external resources or from step six to three processes (see below).

#### 4.1.8. Pipes

These are the basic elements of water transportation.

#### 4.1.9. Digital Contaminant Sensors

These determine the biochemical state of the water by computing factors including temperature, turbidity, oxidation-reduction potential, pH, and conductivity to ensure purity. They can stop the aging of water, the corrosion of pipes, and the entry of contaminants.

Cyber security in water purification and distribution plants is mandatory because the above-mentioned components or elements are linked together, connected to the internet at some point in the system, and are easy targets for attackers without any secure management. There are various stages in a secure water treatment plant, and the PLC is responsible for controlling each stage. Typically, two PLCs are used, with one reserved for emergency use. The general process of securing a water treatment plant is given in the following stages:

- Storage of raw water (received from outside) in a tank;
- In the second stage, chemicals such as HCl, NaCl, and NaOCl are added for better water quality;
- In the third stage, through ultra-filtration, the filtration process starts;
- In the fourth stage, ultraviolet light is used to dechlorinate the water;
- The fifth stage is the reverse osmosis process;
- In the final stage, the remaining water is stored in another tank, and this water can be sent to step three for the backwash process.

### 4.2. Attack Techniques

**Figure 5:**
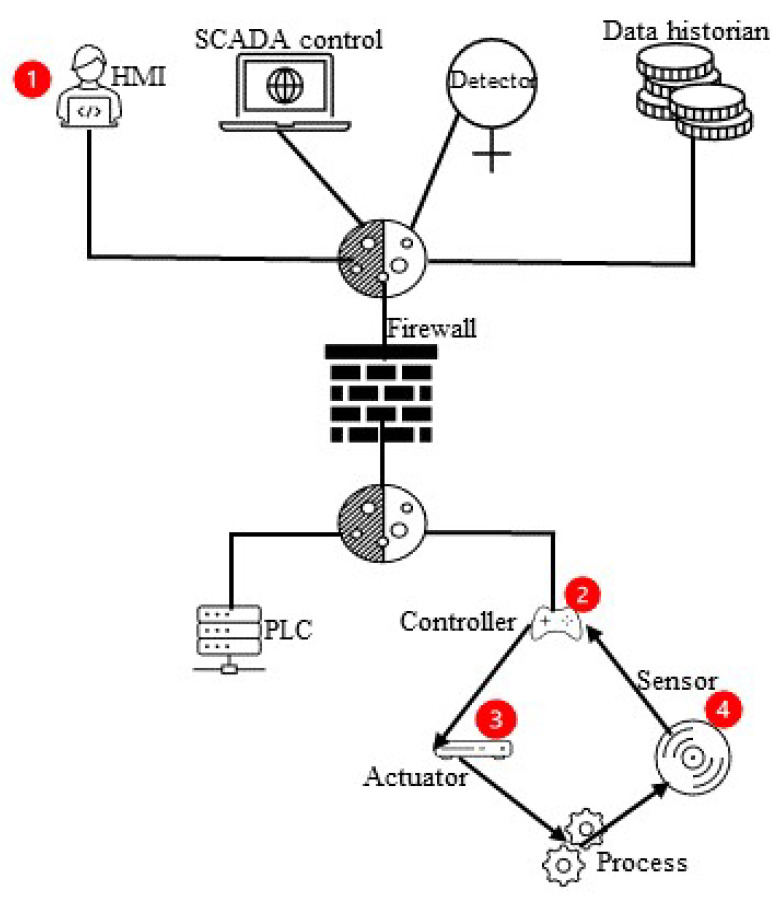
Attacker means of accessing an ICS system.

Water is a basic element of life. It is challenging to secure a water treatment plant, but it is essential not only to secure a plant but to save lives connected with this plant such as those of humans, birds, animals, etc. Advanced and digital technology-based plants are helpful in the improvement of water quality and ensuring a quick supply. However, this deployment of technology also increases the security challenges in the process. For example, an attacker can attack to shut down the water supply, increase the HCl quantity, or overflow the tank. The attacker’s purpose is to damage the plant system. There are several ways that an attacker can attack a water treatment plant, as shown in Figure 5.

#### 4.2.1. Attack through Actuators

In this type of attack, the attacker targets the actuator and provides misinformation concerning the water quantity to disturb the water facilities, as shown in Figure 5.

#### 4.2.2. Attack through PLC

When an attacker targets the PLCs, it leads to the attacker misleading the network operation.

#### 4.2.3. Attack through Sensors

When an attacker attacks through the sensor, it leads to varying sensor [48] readings for private data.

#### 4.2.4. Attack through a Network or Communication Layer

Here, the attacker attacks through the communication or networking links to disturb or damage the system. This communication could be between PLCs, SCADA, sensors, actuators, and the relevant software and hardware used in communication.

The authors of [13] studied all means of maliciously accessing a plant such as through sensors, actuators, networks, and PLCs, etc. In [26,27,30,31,35,36,39], the authors studied attacks through sensors in the environment. Ref. [28] shows an attack via an actuator in the plant. Refs. [31,35] also show means of attack via PLCs’ and actuators’ communication and more than one PLC communication network. The researchers in [35] also represent an attack through PLCs and sensors and the PLC communication link, while [39] only covers attacks through sensors and actuators. Still, there is a lack of security management and the tools necessary for a secure system.

## 5. Attack and Defense Model

The real-world cyber attack cases studied in this review can be grasped efficiently with the understanding of attack and defense models.

### 5.1. Attack Model

The primary objective of the water distribution and purification company is to deliver secure, safe, and pure water to the customers. Because of cyber attacks, there is a danger of disturbances to the water transportation system or variations of chemicals which is a very sensitive issue. A small negligence can cause a big loss. Attackers try to attack through the main components of the plant. From the perspective of the attacker, there are various stages in a systematic process or isolated malicious activity by which to gain access to or reach the target and disturb the network. The killed chain concept has been proposed by researchers in military applications such as weaponization, installation, reconnaissance, delivery command, and control action, etc. It introduced and defined the cyber kill chain (CKC). Taking inspiration from CKC, researchers studied a few attack models.

In an ICS environment, the attack method is quite different because of the different architecture. In an ICS environment, the target can be IT, OT, or DMZ. In real cases, most targets are on the OT network, where attackers gain access to the system or network through the IT network and affect the OT system by applying a number of attacks. This model is known as the combined ICS kill chain and CKS. Here, the attacker can repeat his process to reach the target. He will move inside the network until the time when he accesses the target. Te authors of [49] studied a spiral attack model that moves inside the IT/OT network till it reaches the target. There are various ways for an attacker to enter the ICS environment through sensors, actuators, communication links, or valves, etc. Attacking sensors means varying the water level in the tank. The authors in [50,51] studied an experimental setup to explain the disturbance in the water level in an ICS environment while attacking through actuators to vary the data linked pumping speed and implement a DoS attack. Attacking via the communication link between sensors and PLC means varying the PLC-collected data and getting water status information; attacking via the actuators and PLC communication link means changing the signals and sending wrong information. By attacking via PLCs, attackers can acquire full control of the system and can easily inject a DoS attack; while the attack on sensors means varying the water system configuration system and changing the sensors’ collected data. As shown in Figure 4 the attacker may initiate suspicious activities in an external environment and approach an employee of the organization to insert or inject an attack via email or web server, and a vulnerable host can be helpful to reaching the target. Once an attack happens or is sent into the environment, the attacker is already in the network and moves slowly and safely to reach the target and infiltrate the control or required target. This is an example of a general attack; it is not mandatory that an attack be injected from level 5; an insider can launch an attack, such as an employee in the workstation or DMZ zone. As to how an attacker delivers attacks on victims, the key features are given as follows.

#### 5.1.1. Adversary

It is an organization that is responsible for attacks. It can be from inside or outside and emerge from individual activity or that of a group. In most cyber incidents, this is unknown. It is important to understand the relationship between the attacker and the customer.

#### 5.1.2. Capability

A technique or tool used by the hacker. The target environment’s configuration flaws and vulnerabilities determine an attacker’s capacity.

#### 5.1.3. Infrastructure

A logical or physical communication method such as email or an external device (USB, Hard drive) is used by the attacker to inject the attack and to control the network to attain the required objective. The ICS environment can be monitored, controlled, and attacked by the hacker when he or she injects an attack successfully.

#### 5.1.4. Victims

The system is targeted by the attacker for a self-directed requirement or purpose. The “victims” include any technical issues or vulnerabilities in the network that allow the attacker to penetrate and obtain control of the whole environment. Sometimes, an attacker attains overall control of the ICS environment, and sometimes the attacked gains control of some specific parts, as is often the case in water secure treatment plants; for example, sometimes, the attacker’s purpose is to increase the PH level of water or HCl in the water; sometimes, it is to overflow the tank. For a secure and safe environment, it is important to build a secure algorithm inside the system to detect the attacker’s activity or anomalies before reaching the target. Such an algorithm should also have the capacity to detect the major and minor kinds of anomalies in the world by using various sensors and different dimensional spaces, while there is no need for more sensors in local anomalies. Furthermore, there is a chance that a single attack will hit several sensors at once, confusing the system’s functioning. Here, we study researchers’ contributions to anomaly detection in water purification plants.

The authors of [52] studied nine types of attacks within the water distribution system, and tge attacks studied are through PLCs, SCADA, and sensors. They also studied attack limitations and detection methods. A detection method has been studied in [43] for the identification [53] of local anomalies that affect each sensor individually and worldwide anomalies that use multiple sensors. Researchers in [54] studied an algorithm for the identification of malicious activity by actuators’ regularity and sensors’ data integrity and detected suspicious activities in data. Later on, they separated it from SCADA measurements. The authors of [55] studied intrusion detection for malicious activity in smart cities. The authors detected denial of service attacks (DDoS)—such attacks can disturb the system by flooding the system with a huge number of requests and trying to make it inaccessible to users. Similarly, DDoS disturbs the water purification system by sending a large number of requests. An algorithm for the identification of anomalies consisting of control, consistency, pattern recognition, and hydraulic system has been studied in [56]. For the detection of cyber attacks in water purification plants, model-based fault detection methodology has been studied in [20]. For attack prediction, the authors of [57] presented the long short-term memory recurrent neural network method for testing. There were accurate results in the provided data. In this method, the authors used a limited number of sensors, but it can be enhanced to the overall dataset in the SWaT test bed. Security experts can install several security tools and technologies in an organization for secure processes. Furthermore, experts will have access to databases, standards, benchmarks, and security controls. Safety from security threats is a key challenge in advanced and technology-equipped organizations. A valuable and basic security actions list has been provided by the Center for Internet Security (CIS). The following are the controls suggested by the CIS.

### 5.2. Basic Controls

Basic control consists of an inventory of software and hardware tools, controlled use of admin, and regular vulnerability management. They consist of an inventory of licensed and unlicensed devices and software, their secure configuration, regular vulnerability management, the controlled use of admin privileges, and keeping an eye on audit logs.

### 5.3. Foundational Controls

These are internet-based controls such as email and web browser safety, network secure configuration, malware defence (installation, execution, and spread), and red ream practice. They consist of an information backup process that secures the firewall, wireless access control (track, prevent, and control wireless access), boundary defence (detection and prevention of information flow), data protection, etc.

### 5.4. Organizational Control

These controls involvetraining programs and implementations of security awareness, incident management, and response, red team practice, and penetration tests. In these controls, there are application and software security checks, penetration tests, and red team practice. Controls are available in various security solutions and tools and can have different impacts depending on the target, such as denying or securing the hacker from accessing private information, detecting and attacking, minimizing the attack impact, disturbing or interfering with active malicious activities, containing a consensus zone deceiving the attacker, etc. The authors of [52] studied the various security tools and solutions. Network-based intrusion detection systems, anti-virus solutions, or host-based intrusion detection systems can be useful in the detection process of suspected activities. Malicious activities can be in the trust zone, which consists of various attack steps from delivery to actions and honeypots. Antiviruses are useful in detection and disruption attacks during delivery, and data execution protection techniques are useful in disruption mechanisms.

In old or traditional IT-based security control, there were several OT-based security tools such as unidirectional gateways and data diodes, passive OT intrusion detection, passive tool discovery, and patch management, which are being used in the current ICS environment. It should be noted that each AS is designed for a specific goal but is patched with the OT network, not the IT network. Because several industries moved towards an advanced industry without changing devices or components that were OT patch-managed, there were several vulnerabilities by which attackers could gain access, such as the limitation of network interaction in the unidirectional gateway for IT to OT and the need for a firewall in the new concept of IT and OT, with limited communication regulations limited to the OT network. This is known as boundary defence control, and in the OT network, passive tools discovery is basic control. In a SCADA system, each action requires one or more security controls.

## 6. Evil Eyes on the Water Purification and Distribution Environment in Real-World Incidents

An industrial plant consists of controllers, sensors, and actuators such as robotics manufacturing, power plant controllers, remote telemetry, management systems such as remote terminal units, and the human–machine interference. The interconnectivity of such a complex architecture enables the industrial system to exchange messages or communicate in real time, enhancing system control, providing sufficient real-time awareness, and enhancing efficiency and productivity. In an industrial control system (ICS), devices—opposite to the operating system—are responsible for improving system efficiency, productivity, monitoring, and controlling the system. To meet basic security objectives, ICS device operation requires confirmation of in-transit data, confidentiality of sensed measurement, surety of secure stored information integration, and access only to trusted parties. Confidentiality, integrity, and availability (CIA) are known as the basic principles of security investigation in an ICS sector. Indeed, the confidentiality of stored data in plants is crucial to the ICS sector. In an IIoT automation environment consisting of critical infrastructure such as chemical plants, water plants, nuclear plants, energy plants, etc., since they can result in catastrophic events or uneconomical plant operation, the integrity of the sensed data and the availability of system resources are of the utmost importance. Because of the distinct security objectives of ICS plants and the heterogeneous and interoperable architectures of the ICS sector, comprehensive security research is required in order to to evaluate IIoT security challenges with RWA examples for the best understanding and motivation of incoming researchers. Some of the incidents, their objectives, and limitations are given in Table 2.

IIoT systems combine IT, OT, IoT, and IIoT technologies. So that they may operate effectively in a distributed manner and be monitored and controlled from far-flung areas, IIoT systems can make use of enhanced networking capabilities. This connectivity may cause vulnerabilities; however, in the case of insecure IIoT systems, adversaries can use them to their advantage by breaching industrial networks or distant connections through the Internet. Additionally, attacks can be carried out by accessing compromised industrial staff accounts and abusing their rights. In several industries, attacks have been reported in which either attackers directly attack IIOT systems, such as IIoT device attacks, or they are compromised as a result of the interconnectivity of IT and IIoT systems, in which case attacks travel from IT systems to IIoT devices. In this section, we will discuss some real-world attacks and some lab validations derived from the industrial domain. Figure 3b represents a water purification and distribution plant.

### 6.1. RWA in Water Industry/Plant

Water is an essential component of life. The water and wastewater sector (WWS) is known as one of the most targeted lifeline infrastructures. A large number of cyberattacks in the water industry has been noticed over a short time, and this number is increasing [28]. These cyberattacks disturb water distribution and water treatment/purification systems. In 2020, a large number of cyber attacks was noticed in the water industry [42]. In the water industry in Israel, the first attack was noticed in the modification of the chemicals used to process tap water, followed by attacks on city water pumps and the agriculture sector. Although these attacks were minor and there was no big loss or damage, Israel’s responsible authorities started to focus on strict cyber security measurements in water treatment plants. Israel’s national cyber chief stated “We can see something like this aiming to cause damage to real life and not to IT or data” [42,64].

Out of several incidents in the water industry, one of them is the 2016 attack on a company commonly using the pseudonym “Kemuri Water Company (KWC)” in Israel [14]. After hiring expert security persons in KWC, the company came to learn that there were still many vulnerabilities in their plant architecture. After a careful examination of the KWC systems, the security firm discovered a series of mysterious valve manipulation patterns combined with the IP addresses of state-sponsored hacktivists in the facility’s traffic records and possible unauthorized access to the subsystems. Further investigation helped to collect useful shreds of evidence in the infrastructure of KWC, which uncovered ex-filtration of 2 million plus unique results and chemical flow rate manipulation [43]. The authors of [65] studied a similar water attack incident that happened in 2020. In reality, OT systems were updated according to the times; the OTs used were outdated, and several IT OTs were working with AS400, and a 1988 built-in IBM application system was in use [66]. The following functions were conducted by AS400:

- Controlling flow control applications and several PLC devices for the water valve and the operation of the SCADA platform. The SCADA system in water treatment and purification plant was shown in Figure 3c.
- Billing information of the water utility customers and hosting personally identifiable information. A router for the various KMC-connected networks was used for storing the firms’ financial statements.

In addition, SQL injection vulnerabilities were found in the system [43,67], and the AS400 system was also connected to the Internet. The internal IP address and login information for the web server hosting the payment application was kept in plaintext in a .ini file, giving opponents access. The following vulnerabilities were found by experts in the KWC system:

1. Outdated devices in the systems were in operation;
2. There was no time-to-time updating of the system;
3. The company was using the IBM application system built in 1988 and designed for intermediate and small-sized firms on a large-scale firm;
4. Remote connection vulnerabilities.

Now we would like to summarize the effects of the attack on KWC. There were the following effects: modified the level of chemicals used to process tap water; breaching the personal identification information system; trying to access the customer’s payment information and managing water flow valves and chemical mixtures ratios

Such kinds of attacks are known as “Device Type” attacks because they directly impact the control strategies, limits, and set point of actuators in touch with the industrial processes. After careful investigation, it was suggested that KWC install new technology devices within the system to meet the state-of-the-art security practice for the safety of human lives and the company’s financial interests.

#### KWC Attack Type

**Figure 6:**
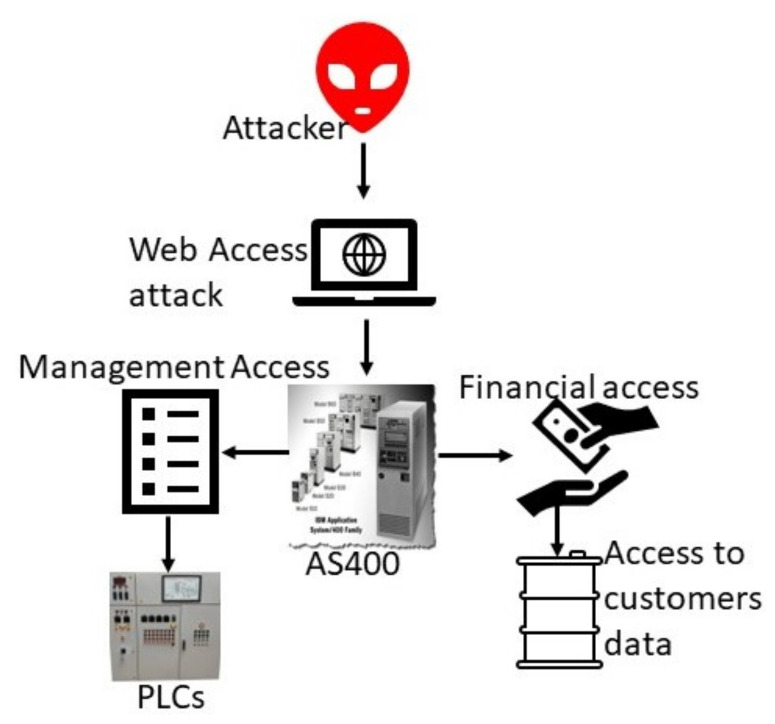
Attacker way to KWC incident.

The attacker found easy access to the login application and breached the personal identification system to obtain access to the client payment information service [67]. Attackers entered using the IT environment, carrying the attack payload to the OT endpoints and damaging the accessible infrastructure and quality of water. This attack proves that industrial facilities, and especially complex infrastructure, must ensure that the security standards and strategies required to meet a secure IIoT system are attained. Figure 6 shows the attacker’s way into KWC.

### 6.2. Florida Water Supply Attack

In [68], there is a new story of a cyber attack on a water treatment plant in a Florida city. A worker in plant management observed that the mouse cursor was moving strangely on the screen and was not in his control, so he informed the local police. In the beginning, there was no concern; his manager frequently connected to his computer to keep an eye on the facility’s systems. The plant utilized the remote-access program TeamViewer to let employees exchange displays and troubleshoot IT difficulties. Within a short time, the attacker was trying to vary the water supply level of sodium hydroxide, moving the setting from 100/ppm (parts per million) to 11,100 ppm. This disturbs the PH level at a low level and damages human tissue it touches at a high level. According to officials [68], the operator quickly overcame the problem and returned the water to its normal position. There is no idea as to how it happened (i.e., whether from inside or outside). The Federal Board of Investigation (FBI) and the city’s own investigation teams are currently searching for the reason behind this attack.

### 6.3. Bay Area Utility Attack for Drinking Water System

Recently, in the US, a hacker obtained access to the Bay Area drinking water system digital network and tried to vary the effluent quality [69]. According to the Northern California Regional Intelligence Center, the attacker gained the TeamViewers login information of one of the company’s former employees in order to obtain access to the system. Refs. [70,71] reported that the hacker tried to poison the drinking water plant serving the San Francisco Bay Area. In initial reports, it was hard to identify the hacker’s means of access. The case will be handed to the Federal Board of Investigation (FBI), who will search for the cause. The Biden government [72] seems interested in overcoming cyber attacks on drinking water plants and other critical infrastructure in the country. It is a good step for the US state to make drinking water safe; however, there a significant challenges, such as the following:

- In the critical infrastructure of the entire country, water treatment plants are considered most vulnerable to hackers. It is difficult for everyone to follow cyber security rules, and it is easy to cause major harm to several people. According to NBC news [70] weather, recently, water plant attacks have increased because there is no specific federal industry for water treatment security accountability.

### 6.4. Colonial Pipeline Attack (CPA) Incident

Sticking to critical US infrastructure, there is the question which type of attack was inflicted om the drinking water system. It has also been confirmed by officials of the FBI that the attacker forced the Colonial pipeline [42] to shut down the gasoline and jet fuel to the East Coast.

The attacker tried to obtain access via ransomware, which is a type of malware that encrypts data until victims, who are threatened with their data being made publicly available via the internet, pay a ransom. Fox News confirmed [71] “In order to prevent the ransomware from spreading and because it was unable to bill customers since it’s business and accounting networks were down, Colonial Pipeline preemptively shut down its pipeline operations”. In Table 3, we present some of the well-known cyber attacks on the water industry from the last five years.

#### CPA Effects

This attack caused the fuel price to rise at a high level. This attack also forced some airplanes to make extra fuel stops and compelled a White House strategy.

### 6.5. Riviera Beach Attack

A police department employee unleashed a devastating ransomware attack on Riviera Beach, a tiny city with 35,000 residents north of West Palm Beach (Florida), on 29 May 2019, openning a compromised email crippling the computer systems of the police force, the municipal council, and other neighborhood government offices, All operations were shut down by the malware, which also encrypted their data. The attack also affected the water utility, compromising the computer systems in charge of pumping stations, testing the water’s purity, and handling payments.

The city council unanimously agreed to allow its insurer to pay the attackers 65 bitcoins, or about USD 600,000, a few days after the attack. The city’s budget would be used to cover an additional USD 25,000 in insurance deductibles. After the attack, two weeks’ investigation revealed that the IT division which created the city’s website and email services was running at full capacity, although the water pump stations and systems for assessing water quality were only partially accessible. The municipal council representative emphasized that although water quality sampling had to be done manually, the water quality itself was never in jeopardy. The FBI, Secret Service, and DHS looked into the incident and advised the city not to pay the ransom.

Instead of waiting for a description key from the attacker, local authorities decided to purchase new hardware and computers by spending USD 900,000 because most of the devices and computers were old and vulnerable to the attacker. This shows that small towns and cities are more vulnerable to attackers because of their old systems.

### 6.6. Secure Water Treatment Plant (SWTP) Lab Validation and Challenges

At the Singapore University of Technology and Design, there is a SWTP test bed plant [50]. This plant has been widely used by researchers [16] for secure mechanisms and control infrastructure. In this section, we studied the introduction and challenges faced by researchers in the lab plant.

#### Introduction of SWTP

SWTP is meeting the latest technology requirements for the water treatment process. Its production capacity is almost 19 liters/minute for water purification by the use of ultra-filtration. This SWTP ICS consists of six stages, with each stage each stage labeled as Pn where n is for the nth stage. At this stage, there are sensors and actuators. Sensors are for measuring the quantity level of water in the tank, the pressure, and the water flow, and also for measuring properties such as conductivity, PH, and oxidation reduction potential. Electric pumps are motor valves which operate as actuators. The functionality of SWTP stages are as follows:

1. Stage 1—Raw water for treatment;
2. Stage 2—Chemical dosing to treat water depending on the measurements from water quality sensors.;
3. Ultrafiltration;
4. Removal of free chlorine from water;
5. Water passes to reverse osmosis process;
6. Cleaning the ultrafiltration unit through the backwash process.

### 6.7. Challenges in Anomaly Detection

Challenges faced during the process of anomaly detection and development are divided into the following categories: model creation; model deployment; and model training.

### 6.8. Challenges in Model Creation

There are following challenges faced during model creation.

1. Supervised vs. unsupervised learning
  - Supervised machine learning (ML) is useful for attack detection [83,84].
  - Supervised ML models are unsuccessful in the detection of unknown attacks due to signature deficiency. The signature updating process is highly complex in ICS.
  - Many researchers and industries prefer unsupervised learning for the process of attack detection [85,86] because unsupervised learning is designed on normal plant operation requirements. A comparison of supervised and unsupervised learning is presented in [85].
  - Unsupervised learning is more useful but can enhance the number of false alarms.
  - Model localization: in a wide system, ICS is a complex distributed control system as there are several steps in the water treatment process that must be connected logically and physically. It is important to consider whether to create a single model for the whole throughout system or for different steps.
  - Scalability: For this, ref. [87] is best work to consult. There is a sensor multitude consisting of pressure, level, quantity, and flow [88]. The work in question shows that unsupervised learning is better than supervised learning for attack detection in water treatment plants.
  - Reliability and data availability.
2. Model deployment is another challenging phase.
  - Data sampling rate;
  - System Modeling: In the system modeling process, modeling the entire physical state space during the training phase is also challenging.
3. Model retraining: Once the detector has been validated, built, and tested in live plant operation, it may demonstrate a different performance than it did in the initial stages, and such observations also face challenges such as distribution shift, noisy data, attack localization, limiting the source attack, unbalanced data, alternation of parameters, process parameter changes, model validation, model complexity, and attack detection speed etc.

## 7. Vulnerabilities, Challenges, and Future Prospective in the IIoT Environment of Water Purification and Distribution Plants

Vulnerabilities assessment in water purification and distribution plants assist in highlighting the malicious activity threat and trigger quick action which can minimize the risk of serious attack from attacker action [43]. This water distribution system vulnerability may impact the overall distribution or purification system [44]. Assessment of vulnerability in a system provides a model by which to reduce the attack impact and reduce the cost. The above studies show that this modern purification plant is linked to open internet and IIoT-linked devices with a number of vulnerabilities, as listed below.

### 7.1. Physical Layer

Vulnerabilities can be due to physical elements such as reservoirs, valves, pipes, motors, PLCs, distillation, tankers, etc. There can be faults or errors in any physical element which may lead to losses [47].

### 7.2. Human Layer

The human is the greatest point of vulnerability for an attacker. Usually, attackers use social engineering methods to enter the environment, and a social engineering attack is conducted via human factors such as employees, managers, and contractors, exploiting a person’s lack of education, greedy nature, or psychological issue [24].

### 7.3. Cyber Layer

SIn CADA and data acquisition etc., some general vulnerabilities are as follows:

- Improper device management (such as installation of devices without any prior knowledge or planning);
- Insecure devices (such as old designed devices which are OT-designed but have no capacity to meet IT requirements);
- Insecure ecosystem;
- Use of old devices/components in a network environment (such as old PLCs, SCADA systems, and OT devices);
- Insecure Network connection (such as communication between sensors and PLCs, actuators and PLCs, or PLCs and SCADA communication).

### 7.4. Challenges and Future Prospective

IIoT is linked with IT and OT. In the above real-world experiments, we studied attacks that happened in the water industry. We also studied the challenges in the real-world water industry. IIoT is not limited to the water industry; it covers all kinds of industries such as energy, chemical, oil and gas, health, agriculture, etc. The authors of [89] explained the means by which to overcome the challenges in water treatment plant security. Here, we would like to discuss some challenges in IIoT or Industry 4.0 [27].

1. Interoperability
  Even if the individual devices in an interconnected system operate in completely diverse fields, they must all be able to communicate with one another and speak the same language. In the world of the IoT, things are made more difficult by a lack of standard software interfaces, data formats, and networking protocols. Due to the incompatibility of various technologies, 40% of the Industrial IoT’s potential economic value will remain locked in the industry. The long lifespan of conventional equipment, which necessitates pricey retrofitting or replacement to work with the most recent technologies, further complicates the goal of seamless interoperability.
2. Security
  The vulnerability to cyberattacks is greater than ever as more and more industrial users demand to be able to remotely access all of their Internet-connected devices and cloud-based services. Cybersecurity has hitherto concentrated on a small set of endpoints. With the introduction of the Industrial Internet, security must broaden its scope to cover both the real and virtual worlds.
3. Reliability
  IP-based networks that are dependable are essential to the IoT’s development. Tried-and-true devices are also essential to a successful network. In the demanding operating environments typical of the oil, gas, maritime, power, and railway industries, device dependability in the IoT is extremely crucial. It is in these sectors’ best interests to install robust enough devices because they will depend more and more on remote access. If the system’s backend is unreliable, even the best-designed and most user-friendly front end will be useless. The adoption of the IoT will be negatively impacted by devices that are flawed or malfunctioning as a result of adverse climatic conditions such as excessive heat or cold. We are all acutely aware of the potential effects of device failure, including risks to individuals, expensive downtime, and incorrect data interpretation, to mention a few. A strong, high-performing backend infrastructure is crucial for the Industrial IoT to succeed.
4. Scalability
  The unpleasant reality is that with billions of additional connected devices on the horizon and billions more now linked, millions of possible event failures are bound to occur. Infrastructures for industrial systems that are scalable and capable of extension are required. By 2020, more than 200 billion “Connected Things” will exist worldwide, according to IDC. Future deployments will also see an increase in mobile, sporadic connectivity, and low-power devices that must interact with a dynamic environment. IoT systems must be scalable and adaptable, using software or additional functionality that seamlessly fits into a larger solution.
5. Network Performance
  Simply put, most networks are not built to handle the problems of an increasing IoT. In order to incorporate video, speech, data, and control commands, integrated networks with high bandwidth are necessary given the trend toward multi-system integration and video surveillance. The limitations of legacy monitoring methods have already been reached. The requirement to collect and analyze an increasingly growing quantity of data points produced by billions of devices will put enormous pressure on networks to expand their bandwidth. In order to move this much data over the Internet, unprecedented amounts of bandwidth are required; otherwise, networks may experience bottlenecks.
6. Management
  Construction of the Industrial IoT must take upgrades and maintenance into consideration. System administrators will be required to oversee all of the new systems as well as the original system as a complex web of interconnected devices grows. By giving network engineers the following tools, modern management solutions simplify their jobs.
  - Application program Iiterfaces (APIs) are an effective tool for streamlining the creation of applications for managing devices or systems;
  - A complete network administration solution that includes setup, running, upkeep, and diagnostics;
  - Instead of relying solely on conventional maintenance schedules, predictive maintenance uses sensors to continuously monitor equipment to avert malfunctions and predict when maintenance will be necessary;
  - Engineers can access real-time device status information and event notifications via mobile management apps;
  - A productivity tool for quickly configuring and deploying devices
7. Future Prospective of IIoT: The IIoT has a bright future ahead of it, with more options for internet-to-device connection and cost-effective predictive maintenance available to small enterprises and multinational organizations. The possibility of connecting more facilities will also increase as IIoT access becomes more affordable. Many experts also believe that WiFi-powered devices will eventually take the place of IoT devices that are currently powered by hardwired connections. Overall, more firms will profit from the IIoT as it becomes more widely available. In the futurem the IIoT will be useful in device management, advancement in sensor technology, predictive IoT gaining adoption, ensuring security, location tracking, keeping an eye on real-time data, IIoT as service, better operational efficiency, wuality control, and customer service. Predictive maintenance, digital twins, cloud edge computing and more connected devices. Furthermore, with the countless advantages of IIoT, experts are also concerned with the increase in cyberattacks. A study [90,91] shows that cybercrime’s global cost will increase from USD 8.44 trillion in 2022 to USD 23.84 trillion by 2027.
  According to [42,92], the Biden administration has vowed to overcome the security issues in the water and oil and gas industries. The U.S. government decided to take fast and quick action against such attacks, which an increase of 50% across the world in 2020 [92]. From the above study, we concluded that water purification and distribution are critical infrastructures; without water, there is no life, and there are serious impacts from cyberattacks on newly deployed water purification and distribution plants. We studied several real-life incidents and attack detection methods. From the study, it has been observed that there are limited attacks, but in the future, this number can increase. Moreover, current attack detection methods are insufficient and are unable to detect all kinds of attacks. Hence, there are several key challenges and research directions that can be addressed by researchers.
  - It has been noticed from the above studies that attack impact can be minimized in purification and distribution plants; however, current research is insufficient to reduce the attack impact. Because of key components such as SCADA, PLCs, and sensors irregularities, faults occur, and these irregularities can cause tank overflow, pipe burst, valve locking, or motor outage. Available water distribution and purification systems deploy certain software and hardware tools to overcome the faults in the system but are limited and expensive.
  - The water distribution and purification plant is a real-time environment and it should not halt its operation, even in a faulty situation. For this, there must be a fault tolerance algorithm or tool that can enhance resilience, separate the damaged or faulty components, and recover from attack effects. For such fault tolerance, deep learning techniques can be useful in attack or fault location identification. Deep learning techniques can also be useful in providing mitigation.
  - It is important to design and develop safe and secure methods. Several studied techniques use deep learning-, machine learning-, or artificial intelligence-based algorithms [93]. However, there are some disadvantages to such approaches, such as ensemble datasets and high dimensional data. Furthermore, the ensemble dataset may cause inaccuracy in the dataset with respect to attack identification when using machine learning techniques. By reducing false negative rates, enhancing data training is also a key issue. The use of hybrid methods is more useful than others. In addition, at time of writing, there has been no work on explaining and detecting the various kinds of attacks in water purification and distribution systems, so it needs more research and attention.
  - IoT devices with various smart devices developed in a purification plant permit transparency in water transportation systems such as monitoring and processing systems. Because of limited resources, the network becomes vulnerable. In this case, privacy and security are both key challenges because of the IoT device’s properties such as less battery timing, low capacity, low memory, and regulations. Violation of privacy reduces the water acceptance level. The key objective of privacy violation is to access private data such as customer data, monitoring data, system functioning data, etc. To date, there has been a lack or deficiency of universal IoT security devices with which to secure the water treatment and distribution plant. To meet the new advanced technology devices, there should be a universal algorithm designed to secure the water supply.
  - For reliability, safety, and security, we require enhancements to the network communication security wit respect to the varying data in various components of the ICS environment. For data exchange, there are heterogeneous networks like wide area networks (WAN), home area Nntworks (HAN), WiFi, etc. This variation in the communication network can become the cause of major cyber attacks in the ICS environment. There are various issues such as verification of heterogeneous networks, key management, access control, and privacy protection. In addition, in smart water purification and distribution plants, there is an exchange of data over the network, and they store data in the network or in the central system such as SCADA, due to which the attacker’s key objective remains targeting the SCADA system to obtain control of the ICS environment. There is a requirement for suitable tools to be installed in a suitable place.
  - Most of the studies show similar attacks and, overall, the work is very limited. For consensus and robustness in water plant security systems, there is a requirement for new, advanced, and updated systems or algorithms that can overcome these issues. Anti-detection attacks, collaborative attacks, and multiple-factor attacks are future research directions in water purification and distribution plants.

## 8. Cyber Safety Tips in IIoT

Everyone understands the importance of water and the water industry in his/her life and of protecting it from digital threats. Many organizations have provided guidelines with which to protect their plants from cyber attacks. The following are some basic guidelines for cyber security protection in IIoT and the water industry [94]:

- Avoid revealing personal or sensitive data to phishers;
- Never click or open any malicious link;
- Prefer to use a VPN when using public WiFi;
- Avoid plugging an unknown USB into your computer;
- Before downloading software, verify its legitimacy;
- Keep software updated;
- Employ up-to-date patches;
- Select prevention over detection technology.

In conclusion, we can say that water purification and distribution plants can be improved via reliability, security, mitigation, detection, and recovery techniques. There should be due consideration of cryptography algorithms such as network communication security, IoT devices security, and cyber–physical design security. At the time of writing this review, there has been very limited work on the security of water purification and distribution plants. In the future, quality research should be conducted in this domain.

## 9. Discussion

There are “Evil Eyes” in the water purification and distribution industry that are creating serious issues for the community, society, and even humanity. It has been noted that these evil eyes are becoming greedy and targeting the industry more and more. The progress of technology is leaving several vulnerabilities in the system and providing gateways to evil eyes through which to observe and enter the system. It has been noted that in traditional or old water distribution systems, evil eyes could only damage the OT infrastructure, while the integration of IT and OT provided an opportunity for these evil eyes to disturb the overall system, vary the chemicals, and can obtain control of the SCADA system.

In this paper we provided some real incidents that occurred because of these evil eyes. We studied system weak point “vulnerabilities” and affected countries and case studies in detail. We also discussed the impact of evil eyes on the environment/system. We explained that evil eyes used advanced technologies such as IT and OT architecture, which are vulnerable to the internet. They are easy targets for evil eyes because of the number of potential objectives to which they are vulnerable, such as getting control of the network, intalling ransomware, varying the chemicals in the water, etc. We feel it necessary to share real case incidents the researchers and industrialists for secure network planning. Most of the cyberattack incidents happened in the USA, while Ukraine, Israel, and the UK are also affected countries. At the time of writing this review, there was little awareness in society regarding evil eye issues and their effects on the water purification and distribution industry.

Most attackers are grouped based on their specific target and objective. Different attacker groups are divided into cyber-terrorists, who cause physical damage to the system; script kiddies, who engage in social engineering attacks such as motivating unskilled labour for entry purposes; hacktivists, who have political or social objectives; and cyber-criminals, who have financial objectives, etc. Researchers also studied [95] a few other groups, such as internal actors, cyber actors, and black hats. The classification depends on the attacker’s objectives and whether the attacker originates from inside or outside the system. We also summarized the attacker’s means of entering the ICS environment.

We provided examples of vulnerabilities in the system, attackers’ target points, and attacker entranceways. We noted that at the time of writing, there was no universal algorithm/technique/mechanism that could secure the water industry from these evil eyes. There is a need for a universal algorithm that could detect, deny, and deceive these evil eyes to protect the plant process. In this paper, we provided the future prospects of cyber security in IIoT and water purification and distribution plants. We also provided some security tips with which to keep the industrial environment secure from these evil eyes.

This survey paper worked on residential, agricultural, and industrial water applications. Studying recent attack incidents and researchers’ efforts, it appears that the development of water monitoring systems to assess the effects of cyber attacks on water quality remains the only focus of research in water and wastewater distribution and purification applications. First and foremost, developing precise, improved detection technologies is crucial. Nowadays, the majority of techniques use artificial neural networks or machine learning techniques. Second, the reliability of data exchange across various WDS components can be increased by strengthening network and communication security. Third, the Internet of Things (IoT) and other intelligent devices provide the water distribution system (WDS) with more utility by ensuring real-time monitoring and automatic processing while allowing transparency in the water supply chain processes. Finally, there is great attention paid to the attack effect in the WDS. In conclusion, measures for mitigation, recovery, and prevention can all improve the WDS. We must take into account both secure software and hardware. Cryptographic algorithms, security network/communication protocols, cyber–physical system designs, and device-level security (such as IoT security), etc., should all be taken into account. According to the literature, there is still a dearth of studies on security for the WDS. Future studies could be conducted on a wide range of subjects.

## 10. Conclusions

The global organized society is currently being impacted, and will continue to be impacted, by the issue of cyber-security. Additionally, as infrastructure becomes more interconnected and communicative, a significant issue with cyber–physical security will arise in key infrastructure, such as the water supply. This paper provided a comprehensive study of the evil eyes in water purification and distribution systems. We discussed real-world cases in the water industry, vulnerabilities, gateways, and evil eye targets. This paper highlighted current real-world cases that occurred in the water and wastewater purification and distribution industry. In this paper, we highlighted how attackers obtain entrance into an ICS system. We covered a number of vulnerabilities and challenges in the system. This paper is useful for securing the ICS environment of water purification and distribution systems and other industries targetted by the evil eye. There are a large number of incidents in the water industry, but we covered only a few of the major incidents for better understanding of readers. We highlighted open challenges in the ICS environment of the water industry and other industries under the threat of evil eyes. We also presented the future prospects of IIoT in an ICS environment and some useful tips. Our paper is intended to promote sufficient knowledge and awareness in the community.

## Figures and Tables

**Table 1:** Comparative analysis with previous related surveys.

R	Y	A	B	C	D	E	F	G
[30,36,45]	2018	✓	x	x	x	x	x	✓
[32,41]	2019	✓	✓	✓	x	✓	x	✓
[37]	2019	✓	✓	x	x	x	x	x
[43]	2020	✓	✓	x	✓	✓	x	✓
[34]	2021	✓	✓	x	x	x	x	✓
[44]	2021	✓	✓	x	✓	✓	x	x
[38]	2022	✓	✓	x	x	x	x	x
[39]	2022	✓	✓	x	x	✓	x	✓
[42]	2022	✓	✓	x	✓	✓	x	✓
[31]	2023	✓	✓	x	x	✓	x	✓
[40]	2023	✓	✓	✓	x	x	x	✓
[33]	2023	✓	✓	x	x	✓	x	x
Proposed Survey	2023	✓	✓	✓	✓	✓	✓	✓

Note: ✓ = Discussed, x= Not discussed, R = Reference; Y = Year; A = IoT; B = IIoT; C = ICS; D = Critical infrastructure (Water and wastewater purification and distribution plants; E = Security; F = System vulnerabilities and challenges; and G = Future prospective).

**Table 2:** Cyber attacks in water and wastewater distribution and purification systems.

Researchers Attack Goals	Gained	Used Methodology	Work Limitations
SCADA, sensors’, PLCs’, and actuators’ communication connections in between PLCs and sensors and in between SCADA and PLCs [52]	Water distribution process had great impact during attack general simulation and algorithm	Simple algorithm	Not useful for attack detection
Communication connection between multiple PLCs and PLCs [58]	Maximum attack detection	Basic detection algorithm	Can be improved by the use of finger-print wireless networks
Actuators and Sensors [55,56,57,59]	Better performance	Anomaly behavior detection algorithm	Time taking
SCADA data [60]	Attack detection ability	Anomaly behaviour algorithm	Incomplete dataset
PLCs, sensors’, and actuators’ communication connections between sensors and PLCs and in between multiple PLCs [54]	Identified maximum attacks	Anomaly detection behaviour algorithm	Less effective data verification algorithm
Actuators and sensors communication link between actuators and PLCs among multiple PLCs [61]	Without delay identification of all labelled attacks	Anomaly detection behaviour	False alarm
Sensors and system communication [62]	Attack detection	Various methods link random forest, multivariate adaptive regression splines (MARS), and neural network	It is not an effective method for malicious detection
SCADA, sensors’, and actuators’ communication [63]	Semi-supervised approach (unsupervised and deep learning)	Better performance and gained maximum accuracy	Incomplete dataset

**Table 3:** List of some famous water industry cyber attacks from 2018–2022.

Year	References	Country	Attack Effect
2022	[19]	UK	Acquired access to bank data
2021	[73]	USA (Southern California)	Acquired control of water treatment plant
2021	[74,75]	Norway	Investigation is ongoing
2021	[76]	Norwegian	Shutdown of water and water treatment facilities in 200 municipalities
2020	[77,78,79]	Israel	Unsuccessful attacks
2020	[80]	Germany	Caused several areas to fail, and operators were unable to shut down a blast furnace properly
2020	[77]	Israel	Raised the level of chlorine in the nation’s water supply
2019	[81]	Kansas (USA)	Obtained access to control measurement
2018	[82]	USA	Disturbed water distribution in critical infrastructure

## Data Availability

No external data are used in this review paper.
